# Glypican-3: A New Target for Diagnosis and Treatment of Hepatocellular Carcinoma

**DOI:** 10.7150/jca.39972

**Published:** 2020-02-03

**Authors:** Meng Guo, Hailing Zhang, Jianming Zheng, Yangfang Liu

**Affiliations:** 1 National Key Laboratory of Medical Immunology &Institute of Immunology, Second Military Medical University, Shanghai, China; 2 Institute of Organ Transplantation, Changzheng Hospital, Second Military Medical University, Shanghai, China; 3 Department of Neurology, Changhai Hospital, Second Military Medical University, Shanghai, China; 4 Department of Pathology ,Changhai Hospital, Second Military Medical University, Shanghai, China

**Keywords:** hepatocellular carcinoma, Glypican-3, immunotherapy, diagnostics

## Abstract

Liver cancer is the second leading cause of cancer-related deaths worldwide, and hepatocellular carcinoma is the most common type. The pathogenesis of hepatocellular carcinoma is concealed, its progress is rapid, its prognosis is poor, and the mortality rate is high. Therefore, novel molecular targets for hepatocellular carcinoma early diagnosis and development of targeted therapy are critically needed. Glypican-3, a cell-surface glycoproteins in which heparan sulfate glycosaminoglycan chains are covalently linked to a protein core, is overexpressed in HCC tissues but not in the healthy adult liver. Thus, Glypican-3 is becoming a promising candidate for liver cancer diagnosis and immunotherapy. Up to now, Glypican-3 has been a reliable immunohistochemical marker for hepatocellular carcinoma diagnosis, and soluble Glypican-3 in serum has becoming a promising marker for liquid biopsy. Moreover, various immunotherapies targeting Glypican-3 have been developed, including Glypican-3 vaccines, anti- Glypican-3 immunotoxin and chimeric-antigen-receptor modified cells. In this review, we summarize and analyze the structure and physicochemical properties of Glypican-3 molecules, then review their biological functions and applications in clinical diagnosis, and explore the diagnosis and treatment strategies based on Glypican-3.

## Introduction

Hepatocellular carcinoma (HCC) is the second leading cause of cancer deaths in the world 1. The HCC incidence cases and number of deaths in China account for more than 50% of the total all over the world, while the mortality rate is in the third place 2. Although the HCC treatment protocols tend to be diversified and combined, it is difficult to achieve further improvement in the long-term survival of HCC patients in the last 10 years. Liver resection or transplantation is the first choice for HCC treatment so far. However, the 1-year recurrent rate is close to 40% after surgical operation, and the 5-year recurrent rate is as high as 50% to 70% 3. Although 5-year disease-free survival rate of patients receiving liver transplantation could reach 60%~80%, 10%~20% of recipients would suffer tumor recurrence after liver transplantation 4. Many studies indicate those tumors have begun to spread circulating tumor cells (CTC) to the bloodstream with a diameter of only 0.5cm-1cm (limit of imaging examination), even forming tiny metastases in distal organs, which become high-risk factors for recurrence and distant metastasis after radical resection in HCC patients 4. Therefore, the search for HCC-specific surface molecules has important clinical significance and application value for the detection, diagnosis and targeted therapy of liver cancer.

Compared with normal hepatocytes, there are many surface molecules high expressed on the surface of HCC cells, such as glypican-3 (GPC3) 5, asialoglycoprotein receptor (ASGP-R) 6, transferrin receptor (TfR) 7, AF20 antigen 8, somatostatin receptor (SSTR) 9, lysosome-associated protein transmembrane 4β (LAPTM4B) 10*.* Among these surface molecules, ASGP-R, TfR, AF20 antigen, SSTR and LAPTM4B are potential therapeutic targets for HCC, but unsuitable for HCC diagnosis. Compared with the those molecules, GPC3 is rarely expressed in adult and not expressed in pathological liver cells such as hepatitis, cirrhosis, and fatty liver. Also, GPC3 located on the surface of liver cells, so that antibody-based drugs or CAR-T/NK can be designed for specific killing of tumor cells with low risk of off-tumor effect.All these features determine GPC3 is the best diagnostic and therapeutic target for liver cancer.

In the past decade, GPC3, which specifically expressed on the surface of HCC cells, has become a new star molecule with high correlation with the occurrence and development of HCC. Not only GPC3 can be used as a biomarker for diagnosis, but also as an important target for immunotherapy of HCC.

Glypican (GPC) belongs to the heparan sulfate proteoglycans family with similar structures, including: a 60-70 kD core protein, which is linked to the surface of the cell membrane by a glycosylphosphatidylinositol anchor (GPI), and the carboxy terminus is modified with a heparan sulfate side chain 11. Currently, there are six members of the GPC family have been identified in the genome of mammals: GPC1 to GPC6 12, 13. All GPCs proteins are highly expressed during embryonic development 14-16: GPC1 is expressed in bone, bone marrow, muscle, epithelium and kidney. GPC2 is specifically expressed in nervous system. GPC3 and GPC6 are widely expressed on various cell surfaces of embryos. GPC4 is expressed in brain, kidney and lung. GPC5 is expressed in brain, lung, liver, kidney and limbs. Compared with embryonic phase, the expression pattern of GPCs changes greatly in adults 15, 17, 18: GPC1, GPC4 and GPC6 are widely expressed in various tissues; GPC2 is no longer expressed; GPC3 is only expressed in the ovary; GPC5 is specifically expressed in the brain.

The expression pattern of GPCs suggests that these molecules play an important role in the growth and morphogenesis during individual development. Recent studies have also shown that GPCs are highly correlated with tumor development. Aikawa, T *et al*. found that GPC1 is associated with pancreatic cancer growth, migration and angiogenesis 19; in addition, Whipple, CA *et al*. revealed that in KRAS mutation-induced pancreatic cancer, GPC1 is the key molecule for tumor growth and angiogenesis 20; exosomes containing GPC1 in peripheral blood can be used as biomarkers for the diagnosis of pancreatic cancer. GPC1 is also up-regulated in breast cancer 21, esophageal squamous cell carcinoma 22, and glioma 23, which suggests a poor prognosis. GPC2 is mainly expressed in the tumors of nervous system, such as neuroblastoma. Yet GPC2 expression in other types of tumors has not been reported. Unfortunately, GPC4, GPC5 and GPC6 are relatively poorly studied. Some studies have shown that GPC4 is highly expressed in pancreatic cancer 24, GPC5 is down-regulated in non-small cell lung cancer 25, and GPC6 is up-regulated in ovarian cancer and positively correlated with prognosis 26.

GPC3 is a star molecule in the GPCs family. Recently, the diagnostic value of GPC3 in HCC has been gradually recognized: GPC3 is specifically expressed in liver cancer tissues, and presents as soluble GPC3 (sGPC3) in peripheral blood of HCC patients, while its expression is not detected in the liver tissues of healthy adults, or pathological samples of fatty liver, or liver with cirrhosis, hepatitis, or injury, suggesting that GPC3 is a more reliable tumor marker than alpha-fetoprotein (AFP). In addition, GPC3 is rarely expressed in other normal tissues of adults, and therefore is suitable for targeted therapy as a tumor antigen 27, 28. Hence, in this review, we summarize and analyze the structure and physicochemical properties of GPC3 molecules, then review their biological functions and applications in clinical diagnosis, and explore the diagnosis and treatment strategies based on GPC3.

## Sequence, structure and physicochemical properties of GPC3

**Figure 1:**
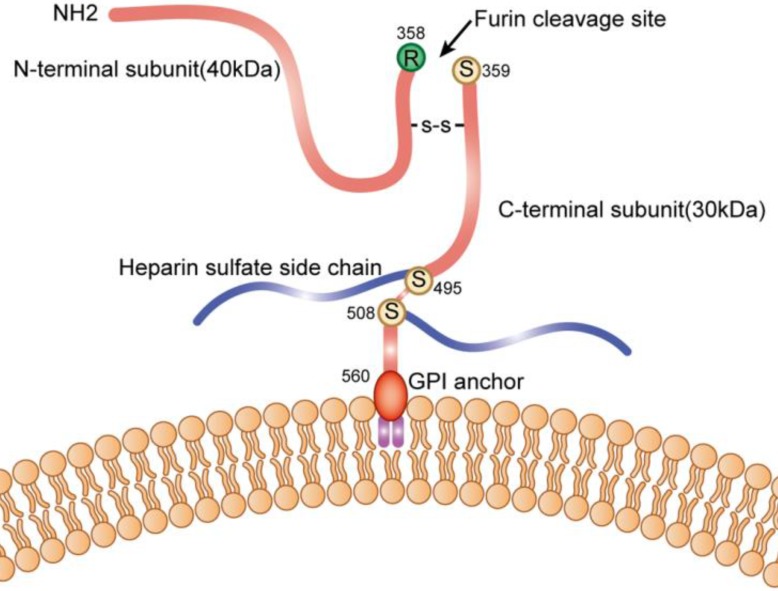
Schematic diagram of GPC3 on the cell membrane.

The GPC3 gene is located on the long arm of the X chromosome at position 26, and contains 11 exons. The transcript is 2130 bp, encoding 580 amino acids, and the molecular weight of the protein is about 70 kDa. The polypeptide contains a Furin restriction enzyme site, and by cleavage of the peptide bond between Arg358 and Cys359, the GPC3 protein is cleaved into two fragments: N-terminal at 40kD and C-terminal at 30kD; These two subunits can be connected by one or more disulfide bonds, and the N-terminal subunit can be further sheared to form sGPC3 in the peripheral circulation; Heparan sulfate modification occurs at two sites of GPC3: Cys495 and Cys508; Ser560 is anchored to the lipid raft on cell membrane by phosphatidylinositol (Figure 1).

**Figure 2:**
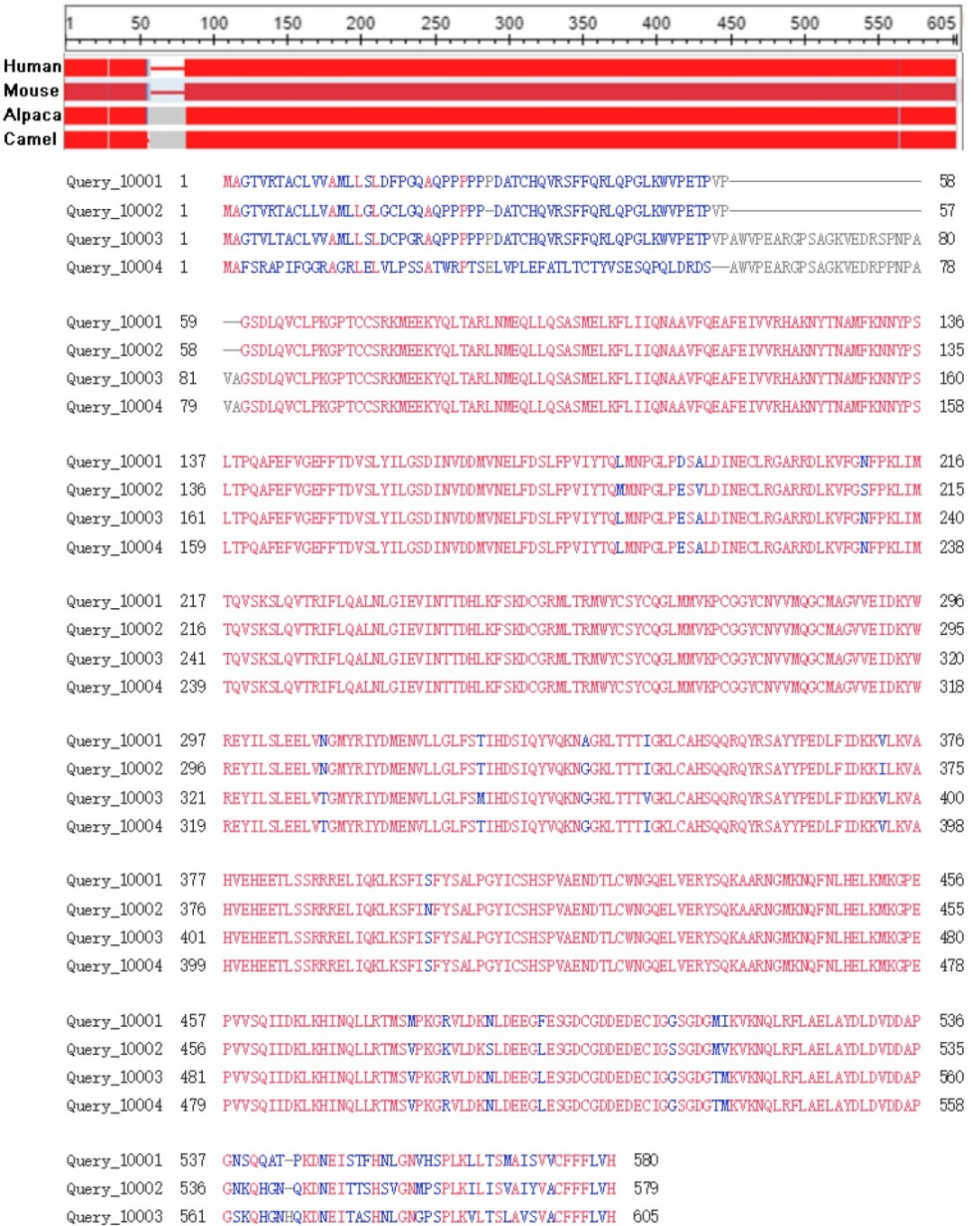
Conservative analysis of the GPC3 protein sequence.

The sequences conservation of GPC3s were analyzed in human (Query_10001), mouse (Query_10002), alpaca (Query_10003), camel (Query_10004) through the in line tool COBALT 29 (Constraint-based Multiple Alignment Tool, https://www.ncbi.nlm.nih.gov/tools/cobalt). The results indicated that GPC3 is highly conservative in these four species (Figure 2), suggesting that direct expression of GPC3 as an antigen may not achieve a good immune effect, and hapten-carrier protein bioconjugate should be used to immunize the animal, or screening the antibody phage display library to obtain an effective antibody.

**Figure 3:**
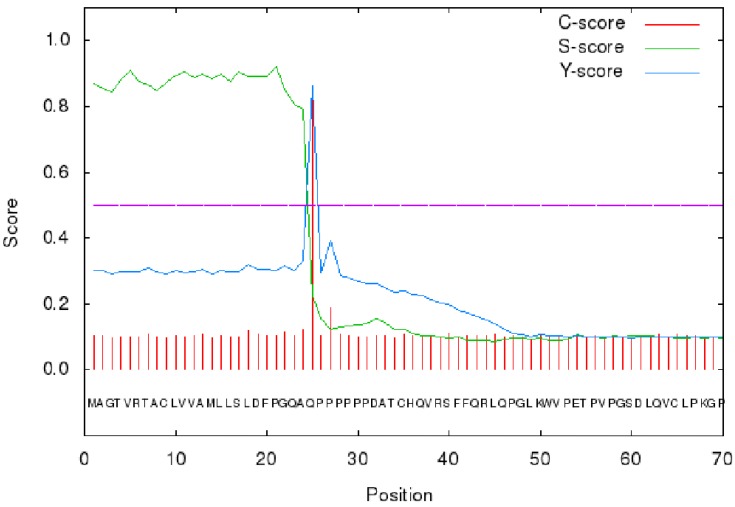
SignalP 4.1 analysis of GPC3 signal peptide.

The signal peptide sequence of GPC3 was analyzed using the on line tool SignalP 4.1 (http://www.cbs.dtu.dk/services/SignalP/) 30. The results showed that the max C and Y scores were located at 25th amino acid, 0.848 and 0.862, respectively, suggesting that the membrane protein signal peptide (Figure 3) consists of the 1-24 amino acids.

**Figure 4:**
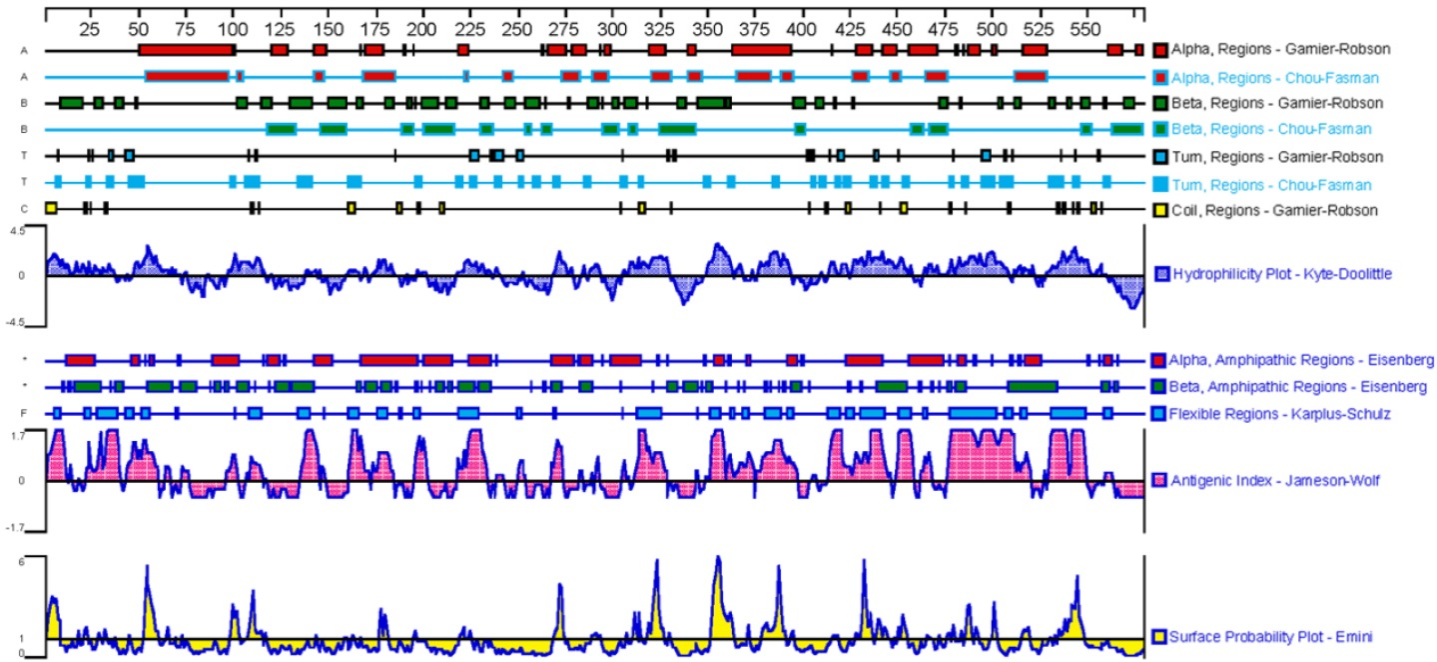
Analysis of the GPC3 secondary structure.

Based on the above analyses, the sequence of GPC3 mature protein was analyzed for physicochemical properties using the on line tool ProtParam (https://web.expasy.org/protparam/). The results showed that the GPC3 mature protein contains 579 amino acids, the molecular formula is C_2950_H_4609_N_779_O_858_S_39_, and the molecular weight is 65967.09Da, the theoretical isoelectric point is 6.01, indicating that the GPC3 protein is acidic. The leucine is the most abundant amino acid in peptide, accounting for 10.3% of the total amino acids. The secondary structure was predicted by DNAstar, as shown in Figure 4. The β-turn and irregular coils, which are often located on the surface of the protein and which is beneficial to antigen-antibody interaction, are richer in the C-terminal subunit. Therefore, the C-terminal subunit shall be used in designing the antigen to immunize animal.

## Biological function of GPC3

Under normal physiological conditions, GPC3 is widely expressed on the membrane of various embryonic cells, but not on those in adult liver. GPC3 mutation causes simpson golabi behmel syndrome (SGBS) in humans, i.e. macrosomia accompanied by dysplasia of multiple organs and skeletons 31. Knockout of GPC3 in mice also causes symptoms similar to SGBS, manifested as excessive growth of somatic cells before and after birth 32. These evidences indicate that GPC3 is involved in the regulation of growth and development of the body.

GPC3 is a membrane protein anchored to the surface of the cell membrane, without an intracellular segment which transmits signals. However, GPC3 is an important component of the extracellular matrix (ECM) 33. GPC3 could interact with a variety of growth factors, chemokines, and cytokines to form a concentration gradient on the surface of the cell membrane, which promotes these ligands binding to their related receptors. In this sense, GPC3 acts as an extracellular signal “recruiter” in various signaling pathways, playing a crucial role in maintaining the concentration of extracellular ligands and promoting ligand-receptor interactions. Studies have shown that the loss of GPC3 during development leads to changes in downstream signals such as WNT and Hedgehog 32, 34-37.

### WNT signaling pathway

**Figure 5:**
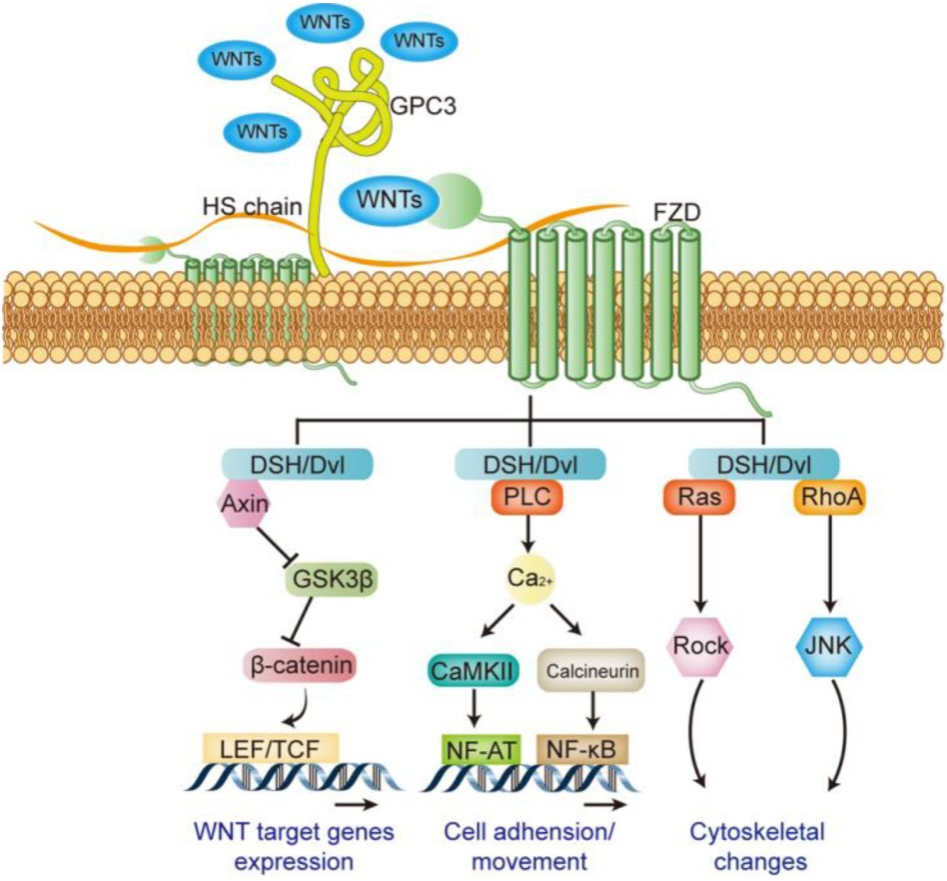
Regulatory effect of GPC3 on the WNT signaling pathway.

WNT signaling pathway plays an important role in many pathological and physiological processes, directly regulating embryonic development, cell differentiation, tumorigenesis and metastasis and invasion etc. The WNT signaling pathway is mainly composed of the ligand WNTs protein family, membrane receptor protein, cytoplasmic signal transduction protein and the downstream transcription factor 38. A total of 18 WNT family members have been identified in humans, including WNT1, WNT2, WNT3, WNT3A, WNT4, and WNT16 etc. Different WNT members can activate different signaling pathways, when WNT1, 2, 3, 3a, and 8 etc. activating their receptors, followed by downstream signal β-catenin, it is called the canonical WNT signaling pathway (WNT/β-catenin pathway). WNT4, 5a, and 5b etc. involve in non-canonical WNT signaling pathways, including WNT/Ca^2+^ signaling pathway and WNT/PCP signaling pathway 38-40. Receptors for WNTs include frizzled (FZD) and LDL receptor related protein5/6 (LRP5/6) 41: the N-terminus of FZD protein carries a cysteine-rich domain consisting of 120 amino acids, and it can bind with high affinity to WNTs 42; LRP5/6 is a single transmembrane protein consisting of about 1600 amino acids, and its extracellular domain is composed of four tandem β-propeller domains. It can bind with different WNTs and form a complex with FZD 43. The membrane complex transmits signals and activates the dishevelled (DSH or Dvl) in the cytoplasm, which in turn mediates canonical or non-canonical activation of the WNT pathway 39 (Figure 5).

GPC3 is an important regulator of WNT signaling pathway in the initial stage. Studies have shown that GPC3 can help the activation of WNT signaling by promoting the formation of membrane surface complexes in liver cancer 44. Both the GPC3 protein and the heparan sulfate side chain can interact with FZD and WNTs 45, 46, and play as a “signal recruiter” in the initial activation-stage of the WNT signaling; in addition, the presence of GPC3 can further stabilize the binding of WNTs to FZD, thereby positively regulate WNTs downstream signal transduction 46 (Figure 5).

### Hedgehog signaling pathway

**Figure 6:**
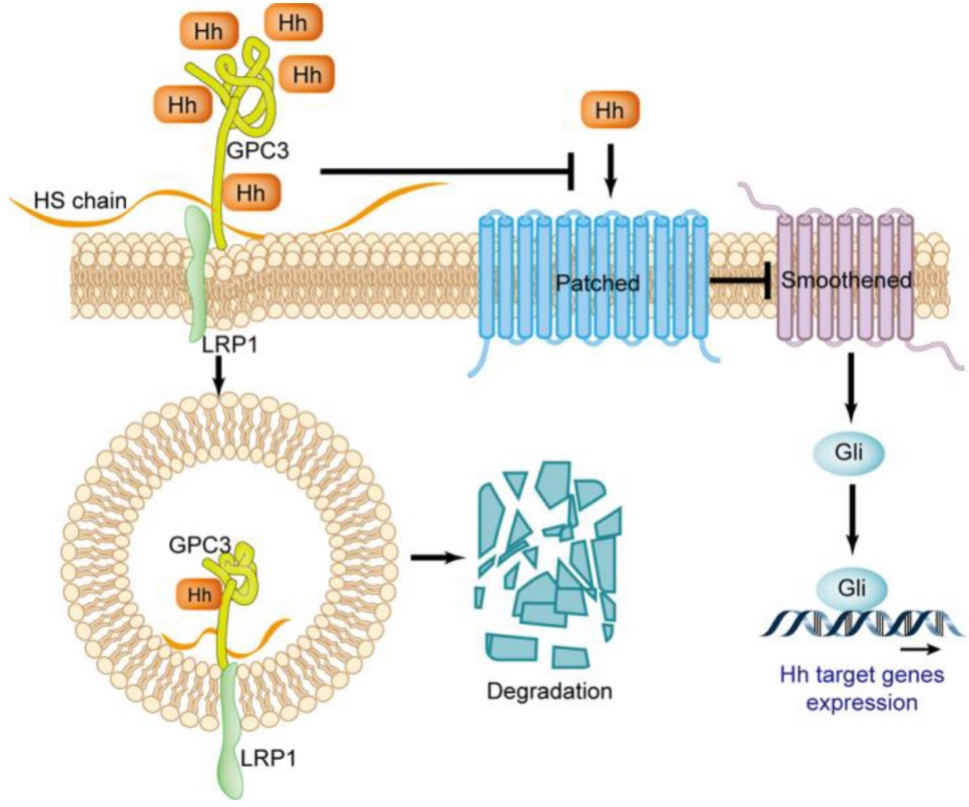
Regulatory effect of GPC3 on the Hedgehog signaling pathway.

The Hedgehog (Hh) signaling pathway plays a key role in embryo morphogenesis, and the abnormal activation of this pathway in adults can lead to the progression of multiple tumors 47-49. Three Hhs have been identified in mammals, including Sonic (Shh), Indian (Ihh) and Desert (Dhh). Shh is widely expressed in various tissues, and Ihh and Dhh are limitedly expressed in a few types of cell 50. The Hh signaling is triggered by the ligand binding to the cell surface receptor celled Patched 47. Patched is a twelve-span transmembrane protein that inhibits the activity of the G protein-coupled receptor family member Smoothened (Smo) before it binds to Hh. When Hh binds to Patched, it will abrogate its inhibitory effect on Smo. The activated Smo triggers the signaling cascade, causing the accumulation of transcription factors Gli1 and Gil2, which in turn regulate the expression of genes for cell proliferation, migration and differentiation 51 (Figure 6).

In the Hedgehog signaling pathway, GPC3 is a potent negative regulator. There is an interpretation about GPC3 loss-of-function mutation resulting in SGBS attributed to excessive activation of the Hh signaling pathway. GPC3 can bind to Shh and Ihh with high affinity, resulting in Hh combined with Patched is drastically reduced, thereby inhibiting Hh downstream signaling 52. The binding of GPC3 to Hh also causes endocytosis and degradation of the GPC3-Hh complex 52, a process that relies on the LRP1 molecule 53 (Figure 6).

### Other signaling pathways

In addition to the WNT and Hh signaling pathways, GPC3 can affect pathological and physiological processes by interacting with other components in the ECM. Midorikawa Y *et al*. showed that the HS side chain of GPC3 can regulate cell proliferation by inhibiting the activity of fibroblast growth factor 2 (FGF2) and bone morphogenetic protein 7 (BMP-7) 54. This inhibitory effect can be relieved by removing the HS chain via heparin-degrading endosulfatase 55. In addition, intervention of GPC3 expression in the HCC cell line promotes TGF-β2 expression, thereby inhibiting cell proliferation 56.

GPC3 also plays an important regulatory role in tumor metastasis. In synergy with HGF/c-Met signaling pathway, the HS side chain of GPC3 can play a role in HCC cell migration. Studies have shown that HS-targeting antibody HS20 inhibits c-Met activation in the treatment of HCC cells, thereby inhibiting HGF-mediated migration and metastasis 57. In addition, studies have shown that GPC3 expression is negatively correlated with E-cadherin in HCC cell lines 58; a similar correlation is also found in pathological examination, and GPC3 overexpression can activate ERK signaling pathway to induce EMT 59.

However, GPC3, as an anchored membrane protein, lacks the relevant structural domain to transmit intracellular signals, and its function on the cell surface is likely to be involved in intercellular or cell-matrix interactions, and its biological function may be far more than those revealed above. For example, Takai H *et al*. confirmed that GPC3 was highly correlated with macrophage recruitment in liver cancer tissues 60. Further studies have found that these macrophages are mainly M2-polarized 61. Therefore, GPC3 may play different roles in different tumor microenvironments, and its biological function may be highly related to the molecular composition in the microenvironment in which it is located. The molecular mechanism of GPC3 needs further exploration.

## GPC3 as a target for clinical diagnosis and its significance

In 1997, Hsu HC *et al*. firstly reported that MXR7 (GPC3) cDNA has a very high positive rate in liver cancer patients 62. Since then GPC3 has become a potential HCC marker, and a large number of studies have been conducted on the expression of GPC3 in HCC tissues and serum. Lots of tissue microarray and immunohistochemical data showed that GPC3 is highly expressed in more than 70% of HCC samples, but not in normal liver tissues, benign liver lesions, liver cirrhosis or hepatitis tissues 5, 63-65. Also, the expression level of GPC3 is correlated with prognosis 66-69. In addition, GPC3 is also highly expressed in some other cancers such as hepatoblastoma 70, lung squamous cell carcinoma 71, ovarian yolk sac tumor 72, melanoma 73, and urothelial carcinoma 74. Therefore, GPC3 is not only a specific biomarker and prognostic factor for HCC, but also a potential target for a variety of tumor treatments.

### Expression of GPC3 in liver cancer tissues

Hsu HC *et al*. firstly reported in 1997 MXR7 mRNA as a potential early HCC marker highly expressed in 74.8% of HCC tissues, and was closely correlated with elevated serum alpha-fetoprotein (AFP) levels (88% *vs* 55%) 62. The MXR7 gene was renamed as GPC3 later. A large number of studies have shown that GPC3 is more sensitive than AFP in the diagnosis of liver cancer. GPC3 is not only expressed on the surface of tumor cells, but also released into peripheral in soluble form (sGPC3). In combination with AFP, we can further improve the sensitivity in non-invasive diagnosis of liver tumors 5, 33, 70, 75, 76.

Studies by Capurro M *et al*. showed that GPC3 was expressed in 72% (21/29) of HCC samples, whereas GPC3 expression was not detected in samples of normal liver or benign liver disease 65. Furthermore, the subcellular location of GPC3 was studied, and results showed that GPC3 is mainly located in the membrane and cytoplasm of HCC cells, and the most common subtype in tumors is isoform 2 (NM_004484.3) 28. As a tumor marker with higher sensitivity than AFP, GPC3 can be used not only for the diagnosis of surgically resected samples, but also for the diagnosis of biopsy samples: Li B *et al*. compared the positive rate of GPC3 in surgically resected samples and that in biopsy samples, and found that the difference between them was small (80.0% *vs* 74.9%) 77.

Although the above studies have confirmed that GPC3 is a specific marker for the diagnosis of HCC, the sensitivity of a single marker cannot meet the requirement for clinical application. Many studies have proposed that HCC can be diagnosed by combination of multiple markers. The best combination of markers is GPC3+HSP70+GS (glutamine synthetase). This combination can specifically distinguish between hepatocellular nodules and early liver cancer and AFP-negative small liver cancer 78, 79. A study confirmed that the specificity of the three-marker combination can reach 100% 80. Other studies have also suggested some potential combinations of markers, such as the combination of GP73, GPC3 and CD34 can improve the specificity of HCC diagnosis to 96.6% 81; the combination of GPC3 and CK19 can be used for differential diagnosis of liver cancer and intrahepatic cholangiocarcinoma (accuracy was 73.5%) 82; GPC3 combined with Arginase-1 and HepPar-1 can further improve the diagnostic accuracy 83.

### Expression of GPC3 in serum of liver cancer patients

As described above, GPC3 can be released from the cell membrane anchored site and then enter peripheral circulation. Therefore, the level of sGPC3 in serum can be an important marker for non-invasive diagnosis of HCC 65. Studies by Capurro M *et al*. showed that sGPC3 was not detected in the serum of healthy donors and hepatitis patients, but 53% of HCC patients had a significant increase in sGPC3 levels 65. Abdelgawad IA *et al* confirmed that the sensitivity and specificity of sGPC3 in serum of HCC patients reached 95% 84. However, it is worth noting that sGPC3 does not have good specificity and sensitivity in all studies. Ibrahim GH *et al* reported that sGPC3 is only 28% positive in HCC patients, far less than the detection rate (80%) in patients with chronic HCV infection. This may be related to the different antibodies used by researchers in different studies. In response to this controversy, Yang SL *et al*. carried out a meta-analysis of 22 studies for HCC diagnosis by sGPC3 levels, and 18 studies showed that serum GPC3 was a specific biomarker for HCC, with a sensitivity and specificity in combination with other markers of 69% and 93% 85, respectively. Therefore, sGPC3 is still an important serum marker for HCC, but it needs to be combined with other markers for joint diagnosis. In addition, large numbers of samples are needed to compare the effectiveness and specificity of different commercial antibodies to determine which antibody is most suitable for clinical diagnosis.

### Prognostic value of GPC3 expression in patients with HCC

Studies by Shirakawa H *et al.* showed that the positive rate of GPC3 in highly differentiated liver cancer was significantly lower than that in moderately and poorly differentiated liver cancer. The 5-year survival rate of GPC3-positive HCC patients was significantly lower than that of GPC3-negative HCC patients (54.5% *vs* 87.7%) 86. In HBV-positive HCC patients, the survival rate of patients with high expression of GPC3 was significantly lower than that of patients with low or no expression of GPC3; and the expression of GPC3 was positively correlated with poor tumor differentiation, portal vein tumor thrombus and tumor lymphatic metastasis 69. For HCC patients complicated with HBV infection /and cirrhosis and undergoing liver transplantation, GPC3 positive indicates a poor prognosis 87. Zhang J *et al*. conducted a meta-analysis of 14 studies covering 2364 patients. The results showed that overexpression of GPC3 may indicate a poor prognosis. The high expression of GPC3 was significantly associated with malignant events such as poor tumor differentiation, advanced stage of tumor, vascular invasion, and HBV infection 66.

### Expression of GPC3 in other cancer tissues

In addition to HCC, there are reports of GPC3 expression in other tumors. The expression of GPC3 is significantly up-regulated in peripheral blood of patients with hepatoblastoma, but its sensitivity and specificity is significantly lower than that of AFP, and has nothing to do with prognosis 70; however, GPC3 can be used as an immunohistochemical marker for hepatoblastoma. A meta-analysis of 4 studies including a total of 134 hepatoblastoma samples showed a positive rate of 95.5% (128/134) for GPC3 88. GPC3 has a higher positive rate in lung squamous cell carcinoma, but has a lower positive rate in lung adenocarcinoma 89-91. A higher positive rate of GPC3 expression in yolk sac tumors (YST), including all YSTs associated with mixed germ cell tumors, it is a key marker for distinguishing between YST and clear cell carcinoma of the ovary (CCC) 72. In melanoma, GPC3 also has a certain positive rate of expression 73, 92, 93, but some studies have shown that GPC3 is negatively expressed in melanoma metastases 94. In urothelial carcinoma (UC), GPC3 has a higher positive rate in malignant UC (43.6% *vs* 13.3%), which is not expressed in normal urothelium. It can be used as a marker when it is difficult to distinguish between low-grade and high-grade tumors 95.

## The treatment strategy of liver cancer with GPC3 as the target

Many studies have shown that GPC3 is specifically expressed on the surface of most HCC cells, but is hardly expressed in other tissues of adults. Therefore, the researchers designed a variety of treatment strategies targeting GPC3 to achieve precise treatment of HCC: antibody-based drugs targeting GPC3, chimeric antigen receptor-modified cells adoptive immunotherapy targeting GPC3, GPC3-related tumor vaccines, antibody-drug conjugate and immunotoxins based on GPC3 antibodies.

### Application of GPC3 antibody in the treatment of liver cancer

GC33 is a monoclonal antibody targeting the carboxyl terminal subunit of the GPC3 molecule. In mouse ectopic and *in situ* GPC3-positive HCC xenograft models, GC33 can significantly inhibit tumor growth 96; its tumor inhibition efficacy is mainly through antibody-dependent cell-mediated cytotoxicity (ADCC) 97. The GC33 humanized antibody is currently the only one that has entered the clinical trial stage 98-100: its phase I clinical trial showed that the GC33 antibody had good safety, and the high dose group did not show significant adverse reactions. The median time to progression in patients with high expression of GPC3 was significantly higher than that in patients with low expression of GPC3 (26 weeks *vs* 7 weeks), and nearly half of the patients were stable (7/13, no progression or remission); however, the results of the phase II clinical trial did not achieve the anticipated goal, in patients with GPC3-positive HCC, treatment with GC33 did not benefit the patients, which may be related to the specific immune microenvironment of liver cancer. This result suggests that antibody therapy targeting GPC3 alone may have limited efficacy.

In addition to GC33, several other therapeutic GPC3 antibodies were screened. Based on the GC33 antibody, Ishiguro T *et al*. have developed a bispecific antibody ERY974 targeting GPC3 and CD3. ERY974 has a good killing effect on a variety of GPC3 high expression tumors, and can convert “cold tumor” into a highly inflammatory state “hot tumor”. It has now entered phase I clinical trials (NCT02748837i) 101. Zhang YF *et al*. identified a YP7 antibody targeting the carboxyl terminal epitope of GPC3, and its scFv-Fc antibody showed better anti-tumor effect in a nude mouse liver cancer xenograft model 102. Feng M *et al*. identified a high-affinity antibody HN3 targeting full-length GPC3. This antibody can inhibit the proliferation of GPC3-positive cells by arresting the cell cycle in G1, and has a significant inhibitory effect on the growth of HCC xenografts in nude mice 103. Gao W *et al*. identified antibody HS20 targeting heparan sulfate chain of GPC3, which blocked Wnt/β-catenin signaling, and inhibited WNT3a-dependent cell proliferation and HCC xenografts growth by blocking the interaction between Wnt3a and its ligand 45. Information on GPC3 antibodies is summarized in Table 1.

### Antibody-drug conjugate and immunotoxins based on GPC3 antibodies

An antibody-drug conjugate (ADC) is a precision therapeutic drug developed based on antibodies, consisting of a monoclonal antibody with specific targeting, a chemically toxic molecule with cytotoxicity, and an adaptor that connects the two. It can concentrate the highly toxic drugs on the tumor site to exert the killing effect, thereby expanding the therapeutic window of small molecular toxins, and significantly reducing the side effects while accurately killing the tumor 104. Based on YP7 antibody, Fu Y *et al*. constructed an ADC coupled with Duocarmycin SA or pyrrolobenzodiazepine dimer - hYP7-DC and hYP7-PC. These two drugs can kill the tumor cells at pMol concentration 105.

An immunotoxin is a pharmacologically active biological preparation constructed from an antibody or a molecular ligand having a specific targeting function coupled to a toxin protein 106, 107. Pseudomonas exotoxin A (PE-A) is the most commonly used toxin fragment in immunotoxins and can cause cell death by inhibiting protein synthesis in cells 106. Several immunotoxins were derived from combination of PE-A and GPC3 antibodies: Gao W *et al*. constructed immunotoxins YP7-PE38 and HN3-PE38, both have good anti-tumor activity *in vivo* and *in vitro*, and can induce regression of GPC3 positive xenografts 108. The same research team truncated the PE-A protein molecule to construct the immunotoxin HN3-mPE24, which also significantly prolonged the survival time of tumor-bearing mice 109. When YP7 scFv identified by Zhang YF *et al*. was coupled with Pseudomonas Exotoxin A (PE38KDEL), it showed a potent anti-tumor effect in a nude mouse xenograft model 102.

Photoimmunotherapy is a new antibody-based biologic agent invented by the Kobayashi team in 2011. It is specifically enriched in carcinoma nest after the near-infrared phthalocyanine dye IR700 coupling with a specific antibody or biomacromolecule, and cell death can be induced by irradiating the drug-bound target cells with near-infrared light 110. The team designed two kinds of photoimmunotherapy agents, IR700-YP7 and IR700-HN3, based on YP7 antibody and NH3, both of which can significantly inhibit tumor growth 111, 112.

### Therapeutic strategies based on chimeric antigen receptors

Chimeric antigen receptor (CAR) refers to a cell surface fusion protein containing an antigen recognition fragment, an immune cell receptor activating molecule, and a costimulatory signal molecule assembled by genetic engineering; after transfecting immune cells (such as NK, T cells, etc.) to express chimeric antigen receptors on the surface, CAR can accurately identify and direct immune cells to kill tumor cells 113. Several groups such as Gao H *et al*. 114, Li K *et al*. 71, Shimizu Y *et al*. 115, and Jiang Z *et al*. 116 constructed CAR-T based on GC33 antibody. These CAR-Ts have good killing effects on GPC3-positive cell lines or PDX. In order to further improve the CAR-T specificity and reduce the risk of off-target, Chen C *et al*. designed GPC3/ASGR1 bispecific CAR-T, which can specifically kill GPC3^+^ASGR1^+^ HCC 117. In order to further enhance the killing effect of GPC3 CAR-T, Pan Z *et al*. co-expressed soluble PD1 (sPD1) fragment with CAR, and sPD1 can protect CAR-T from T cell depletion induced by PD1/PDL1 signaling pathway when co-incubated with target cells 118; Guo X *et al*. found that knockout PD1 in CAR-T by CAS9 can block the downstream signaling of PD1 and significantly enhance the expression of Akt phosphorylation and anti-apoptotic protein Bcl-xL, improving its anti-tumor effect 119. Zhao R *et al*. introduced the intracellular domain of DAP10 into CAR, which significantly improved the killing activity of CAR-T 120. At present, 12 clinical trials have been carried out for the GPC3 CAR-T, as shown in Table 2.

Although CAR-T has a very high response rate in cancer therapy, it also has certain side effects, including off-target effects, cytokine storms and extremely high risk of GVHD. The NK cells, with a short physiological cycle (1~2 weeks) and extensive tumor killing ability, has attracted the attention of researchers. NK cells are a kind of lymphocytes that can kill tumor cells independent on MHC. After recognizing tumor cells, NK cells can cause target cell apoptosis by releasing perforin and granzyme, expressing TNF superfamily members and mediating ADCC. Despite these, compared with T cells, primary NK cells have a series of disadvantages such as limited *in vitro* amplification efficiency and extremely low transfection efficiency. Therefore, many studies have focused on NK-92 cell lines. NK92 is a highly cytotoxic NK cell line that can be continuously and uniformly amplified, and can be applied to establish a stable CAR-NK cell line 121, and has been proved to be excellent in safety by many clinical trials. Nowadays, several CAR-NK products targeting GPC3 have already been developed. Yu M *et al*. constructed a CAR-NK cell line targeting GPC3 based on hu9f2 antibody, which has good killing activity both *in vitro* and *in vivo* 122.

### GPC3 tumor vaccine

A tumor vaccine, which contains a tumor antigen gene or a tumor antigen peptide, can activate acquired immune function to attack tumor cells and prevent tumor growth, metastasis, and recurrence by inducing body-specific cellular immunity and humoral immunity. Tumor antigens include tumor specific antigen (TSA) and tumor associated antigen (TAA). As a typical TSA, GPC3 is specifically expressed on the surface of HCC cells, but is hardly expressed in other cells in healthy adults 123. Therefore, a lot of research results about GPC3 have popped up in the field of tumor vaccine application.

Nakatsura T *et al*. firstly identified a specific GPC3 linear epitope GPC3_298-306_ (EYILSLEEL) peptide in BALB/c mice, which can induce tumor rejection and large numbers of CD8+ T cells into the tumor after xenograft in mice of the tumor cells transfected with this epitope 124; later this group identified the linear epitope GPC3_144-152_ (FVGEFFTDV) peptide, which induces peptide-reactive CTLs in HLA-A2.1 transgenic mice without inducing autoimmunity, and activates CTLs in HCC patients, and these CTLs can significantly kill tumors in the PDX model 125. Based on the above results, this team conducted a phase I clinical trial of GPC3 tumor vaccine in patients with advanced liver cancer 126: 33 patients with advanced HCC were inoculated with GPC3 vaccine, and the results showed that the GPC3 vaccine was well tolerated, and GPC3 specific CTL response was induced in 30 patients. 19 patients were stable 2 months after the start of treatment, and 4 of 19 patients with stable condition had tumor necrosis or regression 127; one 62-year-old sorafenib-resistant liver cancer patient after receiving GPC3 peptide vaccine, most of the intrahepatic tumors had central necrosis, and GPC3-specific CTLs specifically infiltrated the tumor site and did not attack normal liver tissue 128. In addition, the vaccine also achieved good reactivity in clinical trials of GPC3-positive ovarian cancer patients. Two chemotherapy-resistant ovarian cancer patients respectively received GPC3 vaccine injections once every two weeks (6 times in total), a 42-year-old patient suffered advanced recurrent ovarian cancer with liver and retroperitoneal lymph node metastasis showed partial response at 10 weeks after vaccination, and another 67-year-old woman with multiple lymph node metastases remained stable for more than one year 129. At present, the phase II clinical trial of the vaccine for HCC has finished. The recurrent rate of 35 cases of GPC3 vaccine after surgery is lower than that of 33 cases of surgery alone, suggesting that the vaccine has better anti-tumor effect 130.

## Conclusion

Since GPC3 was revealed as a new marker of liver cancer in 1997, its biological function and expression pattern have been gradually identified, and it has evolved from a potential tumor marker to an important indicator for clinical detection, and a series of GPC3 target therapies has been developed. Compared with the traditional HCC marker AFP, the expression pattern of GPC3 anchored on membrane has great clinical transformation potential:

- As a target of *in vivo* imaging, it can accurately locate the liver cancer before treatment, and determine the stage of liver cancer and surgical margins by combining AI technology in image group as well as the non-invasive detection of curative effect and tumor recurrence after surgery;
- Secretory GPC3 provides a reliable new target for early non-invasive screening and diagnosis of tumors;
- Antibody-based drugs derived from GPC3 antibodies and adoptive immunotherapy provide a more accurate and effective way for the treatment of liver cancer.

Despite these, GPC3 still has many technical bottlenecks in clinical applications. The mechanism of GPC3 in the process of liver cancer has not been thoroughly explained. Although it is clearly understood that GPC3 plays an important role in the regulation of WNT and Hedgehog signaling pathways, it is still unclear whether GPC3 can determine the cell differentiation in embryonic development and tumorigenesis. As a membrane-anchored protein, GPC3 lacks the relevant domain that transmits intracellular signals, and its function on the cell surface is likely to be involved in intercellular or cell-to-matrix interactions: GPC3 may be involved in formation of lipid raft rich in cholesterol and glycosphingolipid, the small G protein on the cytoplasmic surface of this lipid raft may be involved in the transduction of GPC3 downstream signaling; in addition, the interaction of GPC3 with other cells in the microenvironment may also regulate important pathophysiology process. It is possible that GPC3 is a “chameleon molecule”, whose biological action is determined by the biological interface of “membrane-matrix”. The molecular composition at this interface determines the biological function of GPC3. These hypotheses also need to be confirmed.

Many previous studies have shown that single anti-GPC3 treatments, including utilization of GC33 or HN3 antibody were not sufficient for elimination of hepatocellular carcinoma. Recombinant immunotoxins and bispecific antibodies developed from GPC3 antibodies have achieved marked therapeutic effects in animal experiments, but their clinical efficacy still need to be verified by clinical trials.

Construction of chimeric antigen receptor-modified immune cells targeting GPC3 seems to be an attractive therapeutic strategy, but it still faces many challenges. Firstly, the microenvironment of liver cancer is always immunosuppressive, such as perfoming high concentration of IL-10 and TFG-β, which directly inhibit the activation of CAR-bearing cells. Secondly, liver cancer, especially advanced liver cancer has special metabolic features, including hypoxia, high lactic acid, nutrient deficiency, etc., which directly leads to dyfunction of immune cells after entering the microenvironment. Finally, some tumors were surrounding by myeloid-derived suppressor cells (MDSC) and T regulatory cells (Tregs), which are known to dampen the immune response and inhibit T cells trying to move into the tumor.

To solve above problems, there are several potential strategies to make anti-GPC3-CAR a true “liver cancer killer”. For microenvironment immunosuppression, the chimeric antigen receptor structure can be furtherly engineered to provide sufficient activation signals. For example, the ligand-recognition domain of IL-10R and TGF-βR can be conjgated to N-terminal of scFv, and then transform suppression signal into activation signal. Moveover, CAR-carriers might be geneticlly engineered to adapt the tumor metabolism. To be specific, CRISPR/CAS9 *in vivo* screening could be performed to find key molecules for the immune cells to survive and activate in a certain metabolic niche. Finally, some studies have shown that several chemical compounds like all-trans retinoic acid (ATRA) had activity in reducing MDSC in tumor nest. The combination therapy with anti-GPC3 CAR-T and compounds like ATRA might further enhance antitumor response.

Subsequently, is the most essential question is that the absence of GPC3 is not lethal to tumor cells. Even if targeting GPC3 strategy could eliminate all GPC3-positive cells, GPC3-negative tumor may grow under such treatment pressure and become resistant. To solve this problem, in addition to finding new tumor therapeutic targets, it is necessary to study the regulation of GPC3 expression to prevent the emergence of drug-resistant clones during the treatment.

## Figures and Tables

**Table 1:** GPC3 related antibodies

Antibody	Affinity	Antigen	Format	Refs.
GC33	0.67 nM	C terminal (524-563)	mAb	96
HN3	0.6 nM	Whole protein	VH+hFc	103
YP7	10nM	C terminal (511-560)	scFv+Fc	102
HS20	0.28 nM	Heparan sulfate	mAb	45
ERY974	0.67nM	GPC3 & CD3	Bispecific antibody	101

**Table 2:** Clinical Application of GPC3 CAR-T (Update to 2018)

Clinic trail	Indication	Status	Phase
NCT02395250	HCC	Completed	I
NCT03084380	HCC	Not yet recruiting	II
NCT02905188	HCC	Not yet recruiting	I
NCT02932956	Pediatric Liver Cancer	Recruiting	I
NCT02876978	LSCC	Recruiting	I
NCT02715362	HCC	Recruiting	II
NCT03130712	HCC	Recruiting	II
NCT03198546	HCC& LSCC	Recruiting	I
NCT02723942	HCC	Completed	II
NCT03146234	HCC	Recruiting	Not Applicable
NCT03302403	HCC	Not yet recruiting	Not Applicable
NCT02959151	HCC	Unknown	II
